# GDC-0349 inhibits non-small cell lung cancer cell growth

**DOI:** 10.1038/s41419-020-03146-w

**Published:** 2020-11-05

**Authors:** Han Yang, Jun Zhao, Mengjing Zhao, Lihao Zhao, Li-na Zhou, Yuxia Duan, Gang Li

**Affiliations:** 1 Department of Chemoradiation Oncology, The First Affiliated Hospital of Wenzhou Medical University, Wenzhou, China; 2 Department of Thoracic Surgery, The First Affiliated Hospital of Soochow University, Suzhou, China; 3 Department of Radiology, The First Affiliated Hospital of Wenzhou Medical University, Wenzhou, China; 4 Department of Radiotherapy and Oncology, Affiliated Kunshan Hospital of Jiangsu University, Kunshan, China

**Keywords:** Non-small-cell lung cancer, Targeted therapies

## Abstract

Non-small cell lung cancer (NSCLC) is a leading cause of cancer-related human mortality with a clear need for new therapeutic intervention. GDC-0349 is a potent and selective ATP-competitive mTOR inhibitor. In A549 cells and primary human NSCLC cells, GDC-0349 inhibited cell growth, proliferation, cell cycle progression, migration and invasion, while inducing significant apoptosis activation. Although GDC-0349 blocked Akt-mTORC1/2 activation in NSCLC cells, it also exerted cytotoxicity in Akt1-knockout A549 cells. Furthermore, restoring Akt-mTOR activation by a constitutively-active Akt1 only partially attenuated GDC-0349-induced A549 cell apoptosis, indicating the existence of Akt-mTOR-independent mechanisms. In NSCLC cells GDC-0349 induced sphingosine kinase 1 (SphK1) inhibition, ceramide accumulation, JNK activation and oxidative injury. Conversely, N-acetylcysteine, the JNK inhibitor and sphingosine 1-phosphate alleviated GDC-0349-induced NSCLC cell apoptosis. In vivo, daily oral administration of GDC-0349 potently inhibited NSCLC xenograft growth in mice. Akt-mTOR in-activation, SphK1 inhibition, JNK activation and oxidative stress were detected in NSCLC xenograft tissues with GDC-0349 administration. In summary, GDC-0349 inhibits NSCLC cell growth via Akt-mTOR-dependent and Akt-mTOR-independent mechanisms.

## Introduction

Over 80% of lung cancer is non-small-cell lung cancer (NSCLC), causing over 1.6 million human mortalities worldwide each year^1,2^. Despite the latest developments in early screening, as well as the adjuvant and neoadjuvant therapies, the average survival for patients with advanced NSCLC is only 8–10 months^1,2^. Personalized molecular-targeted therapies are needed for NSCLC patients^3^.

Due to various gene depletion or mutation (*PTEN*, *PI3KCA*, and *RTK* etc), dysregulation and overactivation of phosphatidylinositol 3-kinase (PI3K)-Akt-mammalian target of rapamycin (mTOR) cascade is detected in NSCLC, which is associated with tumorigenesis and cancer progression^3,4^. Activation of PI3K-Akt-mTOR is vital for cancer cell growth, survival, proliferation, migration, and metabolism, as well as angiogenesis and therapy-resistance. It thus has become an important therapeutic target of NSCLC^3,4^. Recent have tested the anti-NSCLC efficacy of PI3K-Akt-mTOR inhibitors as mono-therapy or in combination with other anti-cancer drugs^4^.

mTOR lies in the central position of PI3K-Akt-mTOR cascade. It is in two multi-protein complexes: mTOR complex 1 (mTORC1) and mTOR complex 2 (mTORC2)^5,6^. mTORC1 is rapamycin-sensitive and composed of mTOR, Raptor, mLST8, PRAS40, DEPTOR, and several others. mTORC1 phosphorylates p70S6K1 (S6K1) and 4E-binding protein 1 (4E-BP1)^5,6^. mTORC2 has several key components, including mTOR, Rictor, Sin1 and mLST8. It serves as the kinase for Akt phosphorylation (at Ser-473) and several other AGC kinases^5,6^. The two complexes are overactivated in NSCLC, emerging as key therapeutic targets.

Conventional mTORC1 inhibitors, including rapamycin and its analogs, only partially inhibit mTORC1 activity without directly affecting mTORC2^7^. mTORC1 inhibition will lead to feedback activation of oncogenic cascades, including PI3K-Akt and ERK-MAPK^8,9^. The second generation of mTOR kinase inhibitors block both mTORC1 and mTORC2, as well as PI3K^7,8^. These agents can completely shut down the whole PI3K-Akt-mTOR pathway, resulting in better anti-cancer activity^7,8^.

GDC-0349 is a potent and selective ATP-competitive mTOR inhibitor^10^. It blocks both mTORC1 and mTORC2^10^. Zhou et al., has shown that targeting mTOR by GDC-0349 potently inhibited head and neck squamous cell carcinoma cell growth^11^. Its potential effect on NSCLC cells, and the underlying mechanisms, have not been studied thus far. Here, we found that GDC-0349 inhibited NSCLC cell growth via Akt-mTOR-dependent and Akt-mTOR-independent mechanisms.

## Materials and methods

### Chemicals and reagents

GDC-0349 was from Dr. Zhou at Hubei Cancer Hospital^11^. Antibodies of phosphorylated (“p”)-Akt (Ser-473) (#9271), Akt (Thr-308) (#13038), Akt1 (#75692), p-S6K1 (#9234), S6K1 (9202), p-JNK1/2 (#9255), JNK1/2 (#9252), SphK1 (#12071), cleaved-caspase-3 (#9664), cleaved-caspase-9 (#20750), cleaved-poly (ADP-ribose) polymerase (PARP) (#5625), and β-tubulin (#15115) were purchased from Cell Signaling Tech (Beverly, MA). All cell culture reagents were obtained from Hyclone Co. (Suzhou, China). N-acetylcysteine (NAC), sphingosine-1-phosphate (S1P) and SP600125, rapamycin, perifosine, AZD-2014, puromycin, and polybrene were purchased from Sigma-Aldrich (St. Louis, Mo). Primers, sequences and all viral constructs were designed and provided by Shanghai Genechem (Shanghai, China) unless otherwise mentioned.

### Cell culture

A549 NSCLC cell line and BEAS-2B lung epithelial cells, both from Dr. Jiang^12^, were cultured as described^13^. Primary human NSCLC cells, derived from three NSCLC patients, “NSCLC-1/-2/-3”, were described in our previous study^13^. The primary human lung epithelial cells were also provided by Dr. Jiang^12,14^. The primary human cells were cultured as describe early^12,14^. Mycoplasma-microbial contamination examination, STR profiling, population doubling time and morphology were checked every 3–4 months to confirm the genotype. The written-informed consent was obtained from each enrolled patient. The protocols of this study were approved by the Ethics Committee of Wenzhou Medical University, in accordance with Declaration of Helsinki.

### Cell viability

Cells were seeded into 96-well plates at 3000 cells per well. Following the applied treatment, cell counting kit-8 (CCK8, Dojindo Laboratories, Kumamoto, Japan) was utilized to test cell viability^15^, and the optical density (OD) absorbance tested at the wavelength of 450 nm.

### Colony formation assay

As reported^13^, A549 cells (at 6 × 10^5^ cells per dish) were re-suspended in 0.5% agar-containing complete medium and added on top of a pre-solidified cell culture dishes. GDC-0349-containing medium was replenished every two days (total five rounds). Cell colonies were counted manually.

### Trypan blue staining

Cells were seeded into six-well plates (8 × 10,000 cells per well). Following GDC-0349 treatment, trypan blue dye was added to stain the “dead” cells, and its ratio was calculated by an automated cell counter (Merck Millipore).

### EdU (5-ethynyl-20-deoxyuridine) assay

Cells were seeded into six-well plates (8 × 10,000 cells per well) and treated with GDC-0349. An EdU Apollo-567 Kit (RiboBio) was applied to examine and quantify cell proliferation. EdU ratio (% vs. DAPI) was calculated from at least 500 cells from five random views under a fluorescent microscope.

### Cell cycle analyses

NSCLC cells were seeded into six-well plates (1 × 100,000 cells per well). Following GDC-0349 treatment, cells were stained with propidium iodide (PI, 10 μg/mL) for 30 min under the dark. FACS was performed to test cell cycle distribution.

### Cell migration and invasion assays

As reported^13,16^, NSCLC cells (in serum free medium, 4 × 10,000 cells per chamber) were seeded at the upper surfaces of “Transwell” chambers (BD Biosciences, Heidelberg, Germany). The lower compartments were filled with FBS-containing complete medium. After 20 h NSCLC cells migrating to the lower surface were fixed and stained. Matrigel (Sigma) was added in the chamber surfaces for in vitro cell invasion assays.

### Apoptosis assays

The detailed protocols for apoptosis assays, including TUNEL staining, Annexin V-propidium iodide (PI) FACS, and caspase-3/-9 activity assays were described in detail in our previous studies^13,17,18^.

### JC-1 assay

In cells with mitochondrial depolarization, the fluorescence dye JC-1 shall aggregate in mitochondria, forming green monomers^19^. NSCLC cells were seeded into six-well tissue-culturing plates at 60% confluence. Following GDC-0349 treatment cells were incubated with JC-1 (10 μg/mL) for 20 min under the dark, washed and tested immediately under a fluorescence spectrofluorometer at 488 nm (Molecular Devices, San Jose, CA).

### Sphingosine kinase 1 (SphK1) activity assay

NSCLC were seeded into six-well plates (8 × 10,000 cells per well) and treated with GDC-0349. The detailed protocols of analyzing SphK1 activity, through ratio-labeled sphingosine-1-phosphate (S1P) spots, were described elsewhere^20^. SphK1 activity was evaluated as pmol/h/g protein.

### Ceramide assay

NSCLC cells were seeded into six-well plates (8 × 10,000 cells per well) and treated with GDC-0349. Cellular pro-apoptotic ceramide contents, in fmol by nmol of phospholipids, were examined by the protocol reported elsewhere^21^.

### Reactive oxygen species (ROS) assay

NSCLC cells were seeded into six-well plates and treated with GDC-0349. Cells were then incubated with CellROX fluorescence dye (10 μM, Invitrogen) for 30 min under the dark at room temperature. CellROX intensity was tested under a fluorescence microplate reader. The representative CellROX fluorescence images were taken as well.

### Glutathione content assay

As reported^20^, NSCLC cells were seeded into six-well plates and treated with GDC-0349. Reduced glutathione (GSH) and oxidized disulfide form glutathione (GSSG) in total cell lysates were tested by a GSH-GSSG assay kit (Beyotime, Wuxi, China). The ratio of reduced to oxidized glutathione (GSH/GSSG×100%) was calculated.

### Western blotting

As described^13,16,17^, an equal amount protein lysates (30–40 μg per treatment in each lane) were separated by 10–12% SDSPAGE gels and thereafter transferred onto PVDF blots. Membranes were blocked and incubated with indicated primary antibodies, and subsequently incubated with corresponding secondary antibodies. The ECL detection reagents (Bio-Rad, Shanghai, China) were then applied to test signals.

### Akt1 knockout

The CRISPR/Cas9-Akt1-KO-GFP construct was provided by Dr. Chen at Jiangsu University^20^. It was transfected to A549 cells by Lipofectamine 2000. GFP-positive A549 cells were sorted by FACS, and monoclonal cells distributed into 96-well plates. Single cells were further cultured in puromycin-containing medium and stable cells established. Akt1 knockout was verified by Western blotting.

### Constitutively-active mutant Akt1

A recombinant adenovirus construct expressing the constitutively-active Akt1 (caAkt1, S473D) was from Dr. Zhang^22^. caAkt1 was transduced to A549 cells. GFP-positive A549 cells were sorted by FACS and monoclonal cells distributed into 96-well plates. The caAkt1 expression in single stable cells was verified by Western blotting.

### Mice xenograft assay

As reported^13^, the severe combined immunodeficient (SCID) mice (18–20 g, all female) were provided by Soochow University Animal Center. A549 cells or NSCLC-1 primary cells (five-million cells per mice, in 200 µL Matrigel-serum free medium) were inoculated subcutaneously to the flanks of SCID mice. Within three weeks xenograft tumors were established with tumor volume close to 100 mm^3^. Mice were then received GDC-0349 administration or vehicle control. The mice body weights and bi-dimensional tumor measurements^23^ were recorded every seven days. The animal protocols were approved by the Institutional Animal Care and Use Committee (IACUC) and Ethics Review Board of Wenzhou Medical University.

### Statistical analysis

The quantitative data in this study were presented as mean ± standard deviation (S.D.). One-way ANOVA plus a Scheffe’ and Tukey Test (SPSS 23.0) were utilized for statistical analyses between different groups. For comparing significance between two treatment groups, a two-tailed unpaired *T* test (Excel 2007) was carried out. *p* < 0.05 was considered as a significant difference.

## Results

### GDC-0349 potently inhibits NSCLC cell viability, proliferation, cell cycle progression, migration, and invasion

**Fig. 1:**
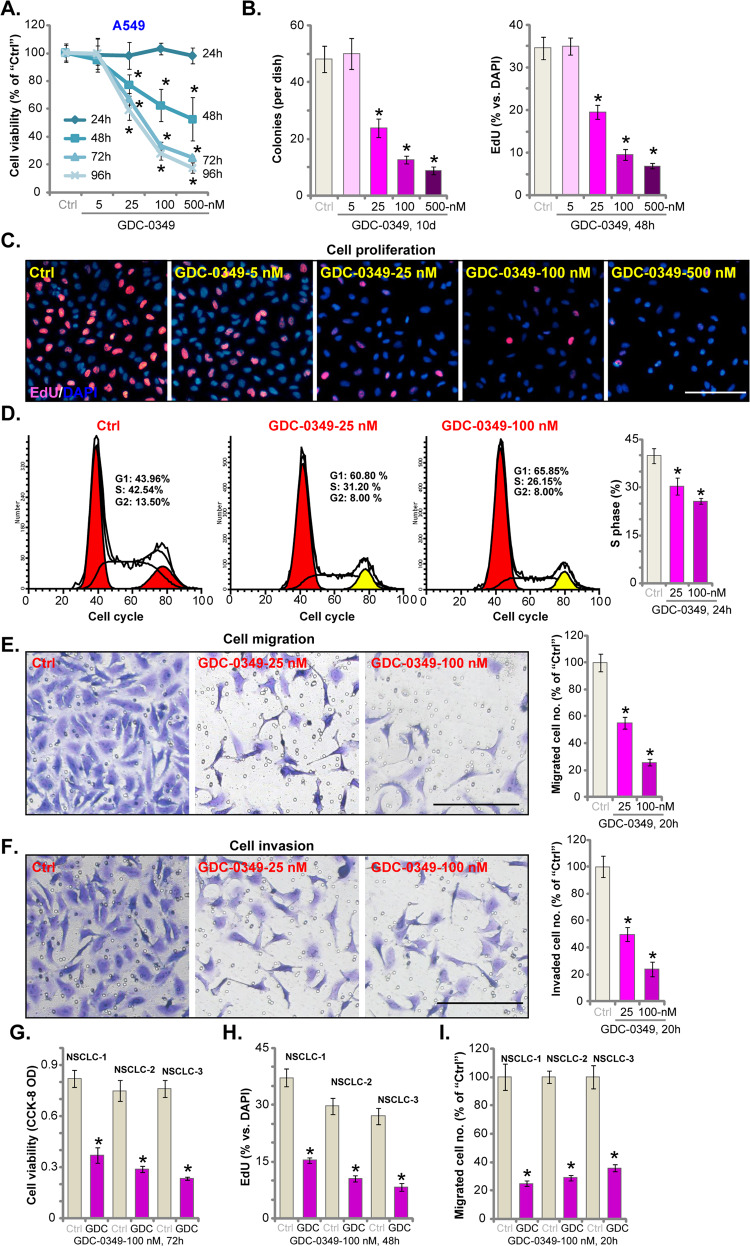
GDC-0349 potently inhibits NSCLC cell viability, proliferation, cell cycle progression, migration and invasion*.* A549 cells (**a**–**f**) or the primary human NSCLC cells (NSCLC-1/-2/-3) (**g**–**i**) were treated with applied concentrations of GDC-0349 (5–500 nM) or the vehicle control (“Ctrl”, same for all Figures), cells were further cultured for applied time periods, cell viability (CCK-8 assay, **a** and **g**), colony formation (**b**), proliferation (nuclear EdU incorporation, **c** and **h**), cell cycle progression (PI-FACS, **d**), migration (“Transwell” assay, **e** and **i**) and invasion (“Matrigel Transwell” assay, **f**) were tested by assays mentioned in the text, and results were quantified. For nuclear EdU staining assays, five random views (1 × 100 magnification) with total 500 cells (for each condition) were included to calculate average EdU ratios (same for all figures). For “Transwell” and “Matrigel Transwell” assays, five random views for each condition were included to calculate the average number of migrated/invaded cells (same for all figures). The data are presented as mean ± standard deviation (SD, *n* = 5). *p < 0.05 vs. “Ctrl” cells. The experiments were repeated five times with similar results obtained. Bar = 100 μm (**c**, **e**, and **f**).

First, A549 lung cancer cells were cultured in FBS-containing complete medium and treated with GDC-0349 (at 5–500 nM). Cells were further cultured for 24–96 h. Testing cell viability, by CCK-8 assays, demonstrated that GDC-0349 inhibited A549 cell viability in a concentration-dependent manner (Fig. 1a). Viability reduction was significant with 25–500 nM GDC-0349 treatment, but being ineffective at 5 nM (Fig. 1a). GDC-0349 also displayed a time-dependent response in decreasing A549 cell viability. It required at least 48 h to observe significant effect (Fig. 1a). Colony formation assay results, Fig. 1b, demonstrated that 25–500 nM of GDC-0349 potently decreased the number of viable A549 cell colonies, further confirming its cytotoxic effect against A549 cells. Testing cell proliferation, by an EdU incorporation assay, showed that GDC-0349 concentration-dependently suppressed A549 cell proliferation, as the EdU-positive nuclei ratio was decreased following GDC-0349 (25–500 nM) treatment (Fig. 1c). At lowest concentration (5 nM) GDC-0349 was again ineffective (Fig. 1c). The IC-50 of GDC-0349 was between 25–100 nM (Fig. 1a–c). These two concentrations were selected for the further experiments.

PI-FACS assay was performed to test cell cycle progression. Results in Fig. 1d demonstrated that in A549 cells GDC-0349 (25/100 nM) decreased S-phase percentage, but increasing G1-phase percentage. These results implied G1-S arrest in GDC-0349-treated A549 cells. The potential effect of GDC-0349 on cell migration was studied next. Using “Transwell” assay, we found that GDC-0349 (25/100 nM) largely inhibited the number of migrated A549 cells (Fig. 1e). Examining cell invasion, by “Matrigel Transwell” assay, we further demonstrated that A549 cell invasion was inhibited with GDC-0349 (25/100 nM) treatment (Fig. 1f). For the migration and invasion assays, NSCLC cells were treated with GDC-0349 for 20 h (Fig. 1e, f), when no significant cytotoxicity was detected (Fig. 1a).

The potential effect of GDC-0349 in primary human NSCLC cells was studied next. As reported in our previous study^13^, NSCLC-1/-2/-3, the primary NSCLC cells derived from three different human patients, were cultured and treated with GDC-0349. As shown, GDC-0349 (100 nM) decreased CCK-8 viability (Fig. 1g), nuclear EdU incorporation (Fig. 1h) and number of migrated cells (Fig. 1i) in NSCLC-1/-2/-3 primary cells. These results demonstrated that GDC-0349 potently inhibited NSCLC cell viability, proliferation, cell cycle progression, migration and invasion.

### GDC-0349 induces NSCLC cell death and apoptosis

**Fig. 2:**
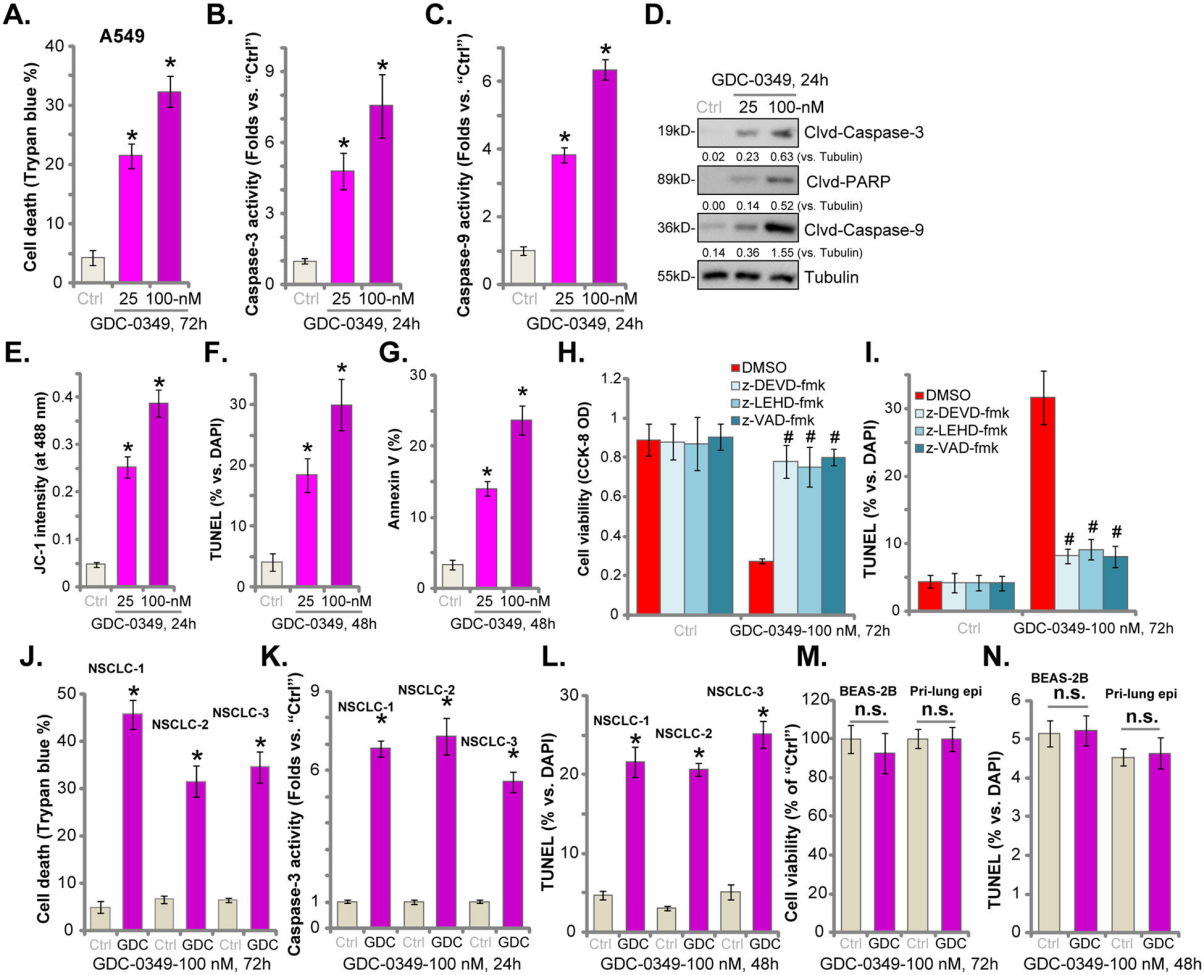
GDC-0349 induces NSCLC cell death and apoptosis. A549 cells (**a**–**g**), the primary human NSCLC cells (NSCLC-1/-2/-3) (**j**–**l**), BEAS-2B epithelial cells or the primary human epithelial cells (“Pri-lung epi”) (**m** and **n**) were treated with applied concentrations of GDC-0349 (25/100 nM), cells were further cultured for applied time periods, cell death was analyzed and quantified by Trypan blue staining assay (**a** and **j**), and caspase activation and apoptosis tested by mentioned assays (**b**–**g**, **k**, **l**, and **n**) with cell viability tested by CCK-8 assay (**m**). A549 cells were pre-treated for 1 h with applied caspase inhibitors (all at 50 μM), followed by GDC-0349 (100 nM) stimulation, cells were further cultured for 48–72 h, when cell viability and apoptosis were examined by CCK-8 (**h**) and nuclear TUNEL staining (**i**) assays, respectively. The data are presented as mean ± standard deviation (SD, *n* = 5). *p < 0.05 vs. “Ctrl” cells. ^**#**^*p* < 0.05 vs. GDC-0349 only treatment (**h** and **i**). “n.s.” stands for no statistical difference (**m** and **n**). The experiments were repeated five times with similar results obtained.

In NSCLC cells proliferation inhibition and cell cycle arrest will result in cell death and apoptosis. Trypan blue staining assay results, Fig. 2a, confirmed that GDC-0349 (25/100 nM, for 72 h) treatment induced A549 cell death, with the number of trypan blue-positive staining cells significantly increased. Activities of caspase-3 (Fig. 2b) and caspase-9 (Fig. 2c) were significantly elevated in GDC-0349 (25/100 nM)-treated A549 cells, where cleavages of caspase-3, caspase-9, and PARP were detected (Fig. 2d). These results implied activation of mitochondrion-dependent cell apoptosis pathway^24,25^. It was further supported by the fact that GDC-0349 induced mitochondrial depolarization in A549 cells, causing JC-1 green monomers accumulation in mitochondria (JC-1 green intensity increase, Fig. 2e). Additional studies confirmed that GDC-0349 induced apoptosis activation in A549 cells, increasing TUNEL-positive cell nuclei (results were quantified in Fig. 2f) and the ratio of Annexin V-gated cells (results were quantified in Fig. 2g). As shown 100 nM of GDC-0349 was again more potent than 25 nM in inducing A549 cell death and apoptosis (Fig. 2a–g).

Next, several known caspase inhibitors were utilized, including the caspase-3 specific inhibitor z-DEVD-fmk, the caspase-9 specific inhibitor z-LEHD-fmk and the pan caspase inhibitor z-VAD-fmk. Co-treatment with the caspase inhibitors largely inhibited GDC-0349-induced viability reduction (Fig. 2h) and apoptosis activation (Fig. 2i) in A549 cells. Therefore, GDC-0349-induced cytotoxicity in A549 cells is due to apoptosis activation.

Similar results were detected in NSCLC-1/-2/-3 primary cancer cells. GDC-0349 (100 nM) induced significant cell death (Fig. 2j), caspase-3 activation (Fig. 2k) and cell apoptosis (nuclear TUNEL ratio increase, Fig. 2i) in primary cancer cells. Conversely, GDC-0349 (100 nM) treatment failed to induce viability reduction (Fig. 2m) and apoptosis (Fig. 2n) in BEAS-2B epithelial cells and primary human lung epithelial cells (“Pri-lung epi”). These results indicated a cancer cell-specific activity by GDC-0349. Together, these results showed that GDC-0349 induced NSCLC cell death and apoptosis.

### GDC-0349 blocks Akt-mTOR activation in NSCLC cells

**Fig. 3:**
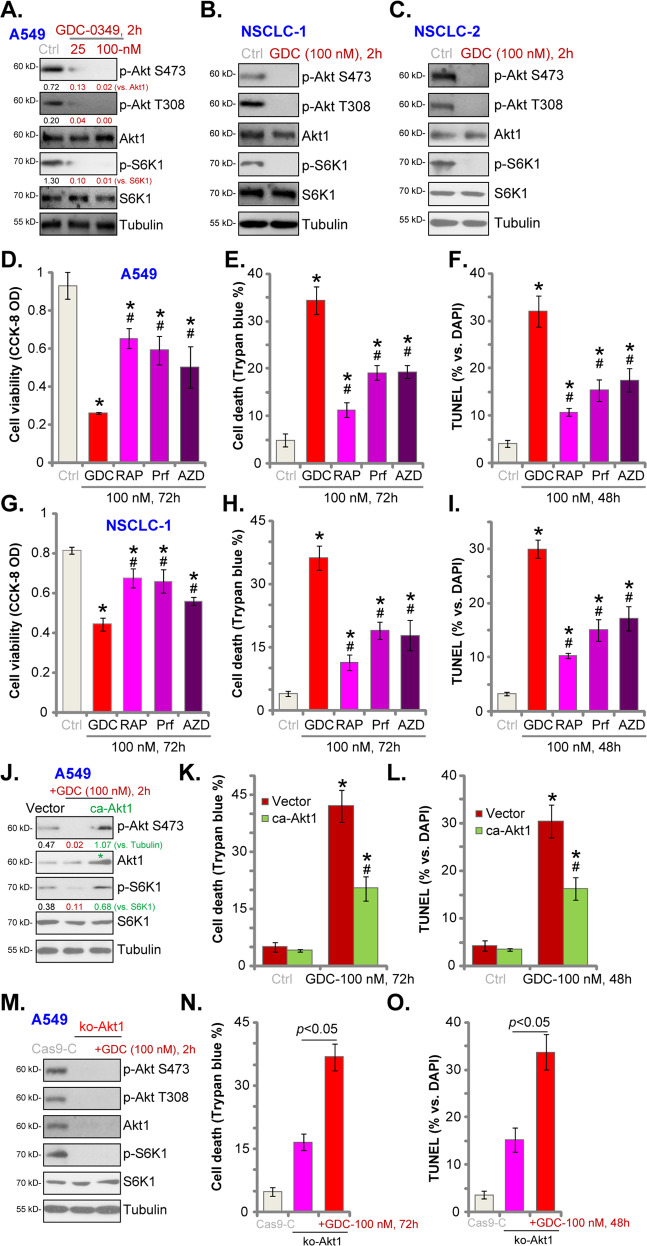
GDC-0349 blocks Akt-mTOR activation in NSCLC cells. A549 cells (**a**) or the primary human NSCLC cells (NSCLC-1/-2) (**b** and **c**) were treated with GDC-0349 (“GDC”, 25/100 nM) for 2 h, expression of listed proteins was tested by Western blotting assays. A549 cells (**d**–**f**) or NSCLC-1 cells (**g**–**i**) were treated with 100 nM of GDC-0349 (“GDC”), rapamycin (“RAP”), perifosine (“Prf”) or AZD-2014 (“AZD”), cells were further cultured for 48–72 h, cell viability, death and apoptosis were tested by CCK-8 (**d** and **g**), Trypan blue staining (**e** and **h**) and nuclear TUNEL staining (**f** and **i**) assays, respectively. Stable A549 cells bearing a constitutively-active Akt1 (ca-Akt1, S473D) or empty vector (“Vector”) were treated with or without GDC-0349 (“GDC”, 100 nM), cells were further cultured for applied time periods, expression of listed proteins was shown (**j**); Cell death (Trypan blue ratio, **k**) and apoptosis (nuclear TUNEL ratio, **l**) were tested. Stable A549 cells with the CRISPR/Cas9-Akt1-KO-GFP construct (ko-Akt1 cells) were treated with or without GDC-0349 (“GDC”, 100 nM), control cells were transduced with CRISPR/Cas9 control construct (Cas9-C); Cells were further cultured for applied time periods, expression of listed proteins was shown (**m**); Cell death (Trypan blue ratio, **n**) and apoptosis (nuclear TUNEL ratio, **o**) were tested. The data are presented as mean ± standard deviation (SD, *n* = 5). *p < 0.05 vs. “Ctrl” cells (**d**–**i**). ^**#**^*p* < 0.05 vs. GDC-0349 only treatment (**d**–**i**). *p < 0.05 vs. “Ctrl” treatment in “Vector” cells (**k** and **l**). ^**#**^*p* < 0.05 vs. GDC-0349 treatment in “Vector” cells (**k** and **l**). The experiments were repeated five times with similar results obtained.

GDC-0349 is a potent and selective mTOR inhibitor^10^. We tested its effect on Akt-mTOR cascade in NSCLC cells. In A549 cells GDC-0349 dose-dependently inhibited phosphorylation of Akt (at Ser-473 and Thr-308) and S6K1 (Thr-389), suggesting blockage of the whole Akt-mTORC1/2 cascade^5,6,26,27^. GDC-0349 treatment (100 nM for 2 h) blocked Akt-S6K1 phosphorylation in primary NSCLC cells, NSCLC-1 (Fig. 3b) and NSCLC-2 (Fig. 3c). Total Akt1 and S6K1 expression was unaffected (Fig. 3a–c).

Next we compared the anti-NSCLC activity of GDC-0349 with other known Akt-mTOR inhibitors, including the mTORC1 inhibitor rapamycin, the Akt specific inhibitor perifosine^28,29^ and mTOR kinase inhibitor AZD-2014^30^. Applying at the same concentration (100 nM) GDC-0349-induced viability (CCK-8 OD) reduction (Fig. 3d), cell death (Trypan blue ratio, Fig. 3e) and apoptosis (nuclear TUNEL ratio increase, Fig. 3f) were more potent than rapamycin, perifosine or AZD-2014 in A549 cells. In NSCLC-1 cells, GDC-0349 was also more significant than these known Akt-mTOR inhibitors in inducing cell death and apoptosis (Fig. 3g-i). These results implied that GDC-0349-induced anti-NSCLC cell activity is not solely dependent on Akt-mTOR blockage.

A constitutively-active Akt1 (ca-Akt1, S473D^22,31,32^) was transduced to A549 cells. Stable A549 cells were established with FACS sorting and puromycin selection. Western blotting assay results, Fig. 3j, confirmed the expression of ca-Akt1 (indicated by the green star). In caAkt1-expressing A549 cells, phosphorylation of Akt and S6K1 was completely restored even with GDC-0349 (100 nM, 2 h) treatment (Fig. 3j). However, caAkt1 only partially inhibited GDC-0349-induced A549 cell death (Fig. 3k) and apoptosis (Fig. 3l). These results further supported the existence of Akt-mTOR-independent mechanisms.

A CRISPR/Cas9-Akt1-KO-GFP construct^20^ was transduced to A549 cells, with stable cells established with FACS-mediated GFP sorting and puromycin selection, named ko-Akt1 cells. Akt1 expression and Akt-S6K1 phosphorylation were completely depleted in the ko-Akt1 A549 cells (Fig. 3m). CRISPR/Cas9-induced Akt1 KO led to cell death (Fig. 3n) and apoptosis (Fig. 3o). Intriguingly in ko-Akt1 cells, GDC-0349 (100 nM) was able to induce further cytotoxicity and apoptosis (Fig. 3n, o). Therefore other mechanisms, besides Akt-mTOR blockage, are responsible for GDC-0349-induced anti-NSCLC cell activity.

### GDC-0349 inhibits SphK1 activation and provokes oxidative stress in NSCLC cells

**Fig. 4:**
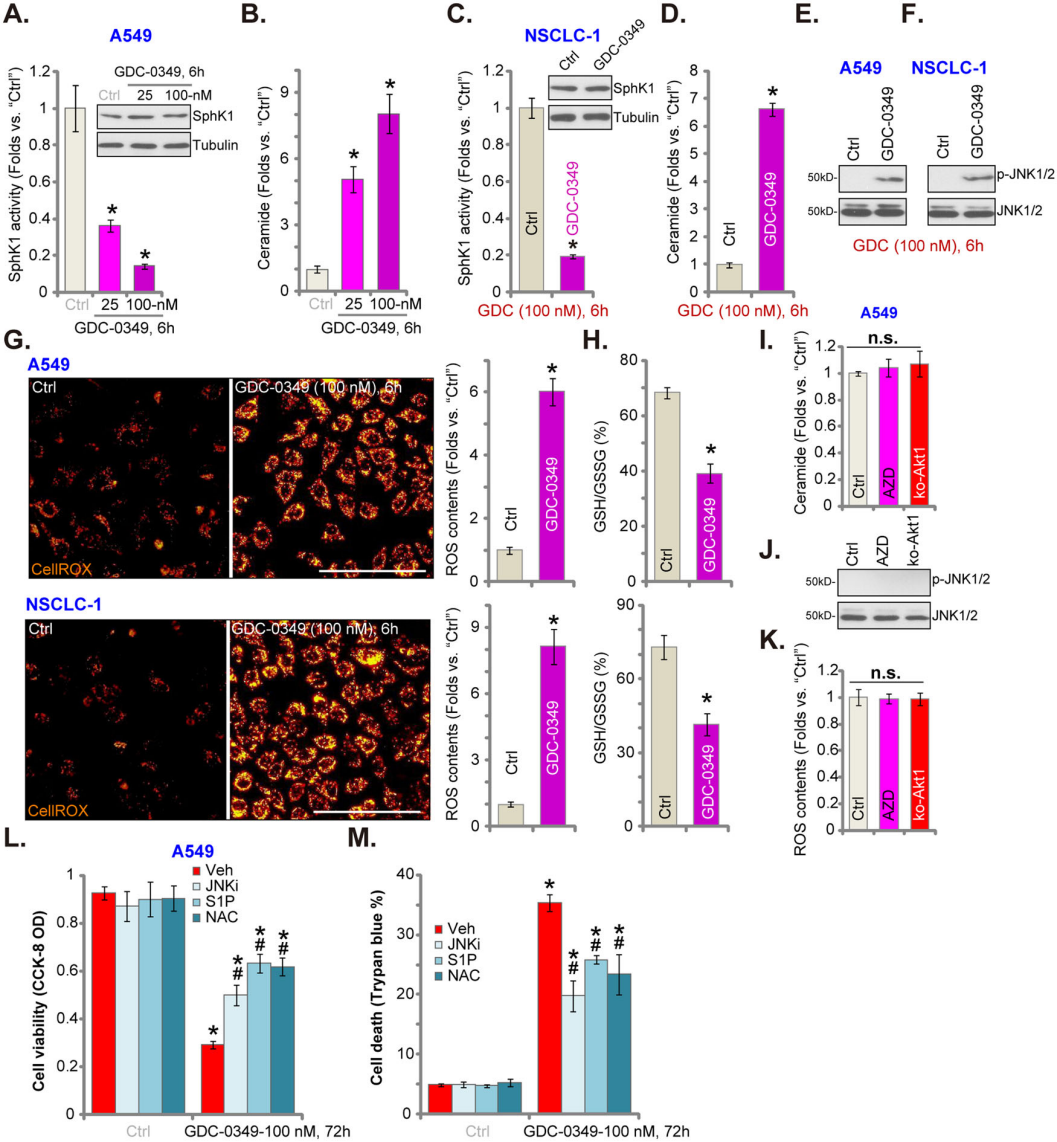
GDC-0349 inhibits SphK1 activation and provokes oxidative stress in NSCLC cells. A549 cells or or the primary human NSCLC cells (NSCLC-1) were treated with GDC-0349 (“GDC”, 100 nM) for 6 h, SphK1 activity and expression (**a** and **c**), ceramide contents (**b** and **d**), JNK activation (**e** and **f**), ROS contents (CellROX intensity, **g**) and the GSH/GSSH ratio (**h**) were tested, and results normalized. Ceramide contents (**i**), JNK expression (**j**) and CellROX intensity (**k**) in A549 cells with or without GDC-0349 (“GDC”, 100 nM, 6 h) treatment, or in stable A549 cells with the CRISPR/Cas9-Akt1-KO-GFP construct (ko-Akt1 cells), were shown, and results normalized. A549 cells were pretreated for 1 h with n-acetylcysteine (NAC, 400 μM), the JNK inhibitor SP600125 (JNKi, 10 μM) or sphingosine 1-phosphate (S1P, 10 μM), followed by GDC-0349 (100 nM) stimulation, cells were further cultured for 72 h, cell viability (CCK-8 assay, **l**) and death (Trypan blue ratio, **m**) were tested. The data are presented as mean ± standard deviation (SD, *n* = 5). *p < 0.05 vs. “Ctrl” cells. ^**#**^*p* < 0.05 vs. “Veh” (0.2% of DMSO) group (**l** and **m**). The experiments were repeated five times with similar results obtained. Bar = 100 μm (**g**).

SphK1 is an important oncogene for tumorigenesis and progression of NSCLC^33^ and other cancers^34,35^. We therefore analyzed whether GDC-0349 could affect SphK1. SphK1 activity assay results, Fig. 4a, demonstrated that GDC-0349 (25/100 nM, 6 h) inhibited SphK1 activity in A549 cells. Consequently, levels of the pro-apoptotic ceramide, which can be accumulated with SphK1 inhibition^36–38^, were elevated (Fig. 4b). In NSCLC-1 primary cells, GDC-0349 suppressed SphK1 activity (Fig. 4c) and induced ceramide accumulation (Fig. 4d). Expression of SphK1 was unchanged (Fig. 4a, c). Ceramide accumulation could provoke JNK activation to promote cell apoptosis^39–41^. In A549 cells and primary NSCLC-1 cells, JNK1/2 phosphorylation, indicating JNK activation, was significantly increased with GDC-0349 treatment (Fig. 4e, f).

ROS production could be another important mechanism responsible for cancer cell apoptosis with treatment of anti-cancer drugs^42–44^. We therefore tested whether GDC-0349 altered ROS contents in NSCLC cells. Using a CellROX dye assay^45,46^, we showed that cellular ROS levels were significantly elevated following GDC-0349 treatment in A549 cells and NSCLC-1 cells (Fig. 4g). GSH/GSSG ratio was decreased (Fig. 4h), further confirming oxidative stress in GDC-0349-treated NSCLC cells. Importantly, AZD-2014 (the mTOR kinase inhibitor) and CRISPR/Cas9-induced Akt1 KO (see Fig. 3) failed to provoke ceramide accumulation (Fig. 4i), JNK1 activation (Fig. 4j) and ROS accumulation (Fig. 4k) in A549 cells. Therefore, these are the unique actions by GDC-0349 and are independent of Akt-mTOR blockage.

Functional studies demonstrated that the ROS scavenger NAC, the JNK inhibitor SP600125 and anti-ceramide lipid sphingosine 1-phosphate (S1P)^47^ alleviated GDC-0349-induced viability reduction (Fig. 4l) and cell death (Fig. 4m) in A549 cells. Therefore, SphK1 inhibition, JNK1 activation and ROS production, independent of Akt-mTOR blockage, contributed to GDC-0349-induced anti-NSCLC cell activity.

### GDC-0349 oral administration inhibits NSCLC xenograft growth in SCID mice

To study the potential anti-NSCLC activity of GDC-0349 in vivo, A549 cells were subcutaneously (*s.c*.) injected to the flanks of SCID mice. Within three weeks A549 xenograft tumors were established and tumor volumes close to 100 mm^3^ (labeled as Day-0). A549 xenograft-bearing SCID mice were then randomly assigned into three groups, receiving GDC-0349 (10 or 30 mg/kg, daily, oral administration) or the vehicle control.

**Fig. 5:**
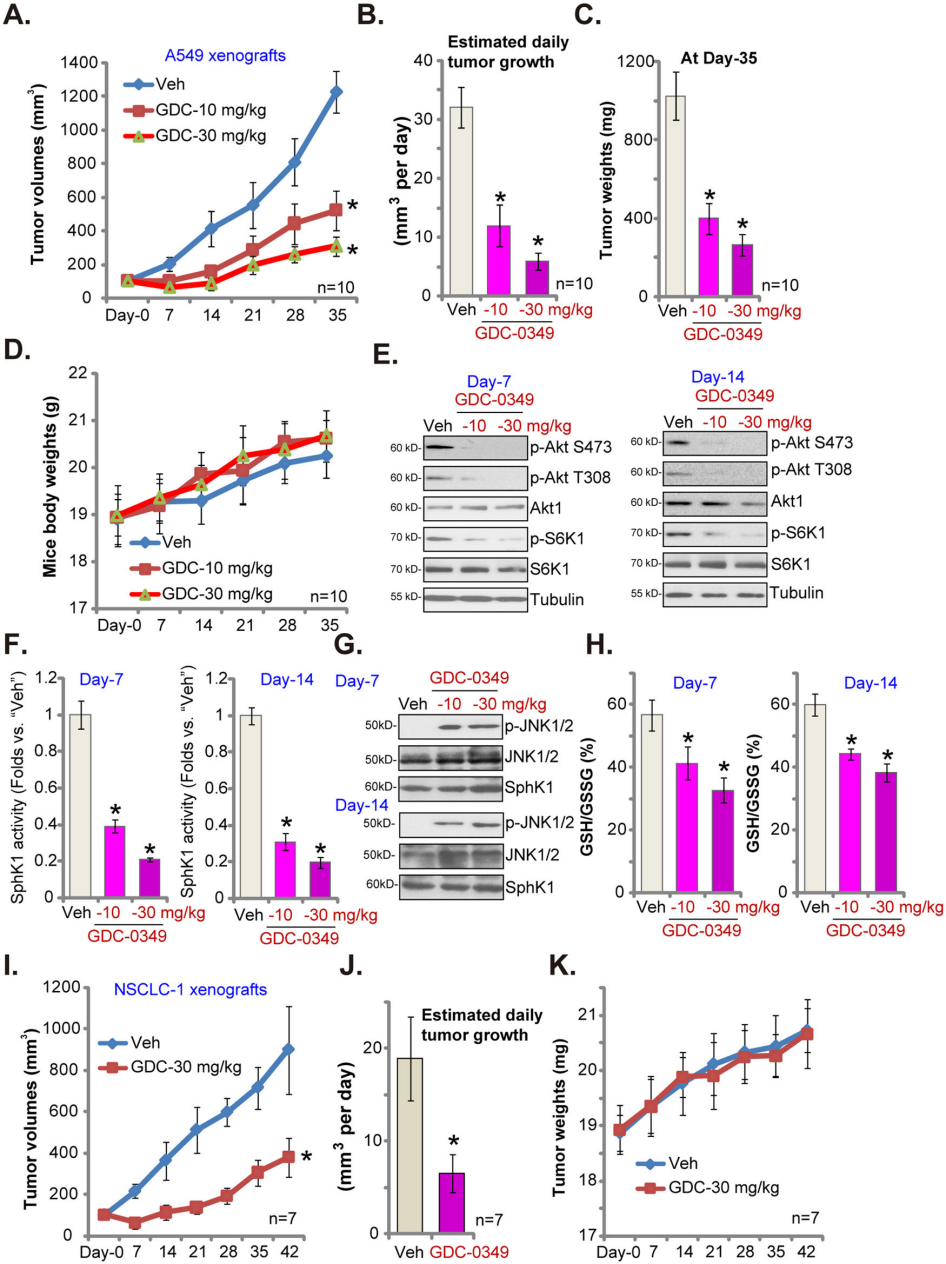
GDC-0349 oral administration inhibits NSCLC xenograft growth in SCID mice. A549 xenografts-bearing SCID mice were randomly assigned into three groups (10 mice per group); Mice received GDC-0349 treatment (oral administration, 10 or 30 mg/kg body weight, daily for 21 days) or vehicle control treatment (“Veh”); Tumor volumes (**a**) and mice body weights (**d**) were recorded every seven days. Estimated daily tumor growth was calculated using the described formula (**b**); Tumors of all three groups were isolated and weighted at Day-35 (**c**). At Day-7 and Day-14, one tumor from each group was isolated (total six tumors), and tumor lysates achieved; Expression of listed proteins was shown (**e** and **g**); Relative SphK1 activity was tested (**f**),with GSH/GSSG ratio examined as well (**h**). NSCLC-1 xenografts-bearing SCID mice were treated with GDC-0349 (oral administration, 30 mg/kg body weight, daily for 21 days) or vehicle control; Tumor volumes (**i**) and mice body weights (**k**) were recorded every seven days. Estimated daily tumor growth was calculated by the formula: (tumor volume at Day-42—tumor volume at Day-0)/42 (**j**). The data are presented as mean ± standard deviation (SD). *p < 0.05 vs. “Veh” group.

Tumor growth curve results, in Fig. 5a, showed that GDC-0349 administration potently inhibited A549 xenograft growth in SCID mice. Volumes of A549 xenograft tumors in GDC-0349-treated mice were significantly lower than those in vehicle control mice (Fig. 5a). GDC-0349 at 30 mg/kg was significantly more potent than 10 mg/kg in suppressing A549 xenograft growth (Fig. 5a). The estimated daily tumor growth was calculated by the formula: (tumor volume at Day-35—tumor volume at Day-0)/35. Results demonstrated that A549 xenograft growth was largely inhibited by GDC-0349 administration (Fig. 5b). At Day-35, all xenografts were carefully isolated from the mice and weighted individually. Results showed that A549 xenografts in GDC-0349-treated mice were much lighter than those in vehicle control mice (Fig. 5c). The mice body weights were not significantly different between the three groups (Fig. 5d). We failed to identify any apparent toxicities in experimental animals, indicating that mice were well-tolerated with the applied GDC-0349 treatment.

To test signaling changes in vivo, at Day-7 and Day-14 one tumor from each group was isolated, total six tumors were homogenized and tumor tissue lysates were achieved. Western blotting assay results, Fig. 5e, demonstrated that phosphorylation of Akt and S6K1 was largely inhibited in GDC-0349-treated A549 xenografts (Fig. 5e). Furthermore, GDC-0349 administration inhibited SphK1 activity in A549 xenografts (Fig. 5f) but provoked JNK activation (Fig. 5g). Total SphK1 expression was unaffected (Fig. 5g). Further analyses demonstrated that the GSH/GSSG ratio was deceased in A549 xenograft tumor tissues with GDC-0349 administration (Fig. 5h), suggesting that GDC-0349 induced oxidative injury in vivo. Therefore, the in vivo signaling changes were in line with the in vitro findings. While blocked Akt-mTOR activation, GDC-0349 also induced SphK1 inhibition, JNK1 activation and oxidative injury in A549 xenografts.

Alternatively, the primary NSCLC-1 cells were injected *s.c*. to the flanks of SCID mice. Xenograft tumors were established within three weeks. As shown oral administration of GDC-0349 (30 mg/kg, daily for 21 days) potently inhibited NSCLC-1 xenograft growth in SCID mice (Fig. 5i). Calculating estimated daily tumor growth demonstrated that NSCLC-1 xenograft growth was again slowed by GDC-0349 administration (Fig. 5j). The mice body weights were not significantly different between the two groups (Fig. 5k). Therefore, GDC-0349 oral administration inhibited NSCLC xenograft growth in SCID mice.

## Discussion

PI3K-Akt-mTOR cascade is vital for cell viability, growth, proliferation, metabolism, and migration^48–50^. This cascade is commonly dysregulated and overactivated in NSCLC^4,51^. The pharmacological inhibitors of this cascade have been intensively tested for anticancer therapy^4,51^. mTORC1 inhibitors, including everolimus and temsirolimus, have been approved by FDA for the treatment of certain advanced renal cell carcinoma^52,53^ and breast cancer^54,55^. However, mTORC1 inhibitors can only partially inhibit mTORC1 activation without directly suppressing mTORC2^54,55^. Rapamycin and its analogs could induce feedback loop activation of oncogenic cascades, i.e., PI3K-Akt and Erk-MAPK^54,55^. Studies have shown that inhibiting PI3K or Akt alone will also result in activation of upstream receptor tyrosine kinases^48,49,56^. It is therefore necessity of simultaneous targeting of multiple signaling cascades for efficient anticancer therapy for NSCLC^4,51^.

GDC-0349 is a potent and selective ATP-competitive mTOR inhibitor. In this study, we reported that GDC-0349 blocked Akt-mTORC1/2 activation in NSCLC cells. It potently inhibited NSCLC cell growth, proliferation, cell cycle progression, migration and invasion, and simultaneously inducing apoptosis activation. It is however non-cytotoxic in lung epithelial cells. Daily oral administration of GDC-0349, at well-tolerated doses, potently inhibited NSCLC xenograft tumor growth in SCID mice. Akt-mTOR inhibition was detected in GDC-0349-treated xenograft tumor tissues.

Combined targeting of PI3K-Akt-mTOR pathway along with other signaling cascades in NSCLC cells have provided promising preclinical results, which should be much better than single PI3K-Akt-mTOR blockage^4^. Although GDC-0349 blocked Akt-mTORC1/2 activation, we suggested that GDC-0349-induced anti-NSCLC cell activity was not solely dependent on Akt-mTOR blockage. First, GDC-0349 was significantly more potent than other known Akt-mTOR inhibitors (rapamycin, perifosine and AZD-2014) in inducing NSCLC cell death. Second, restoring Akt-mTOR activation by caAkt1 only partially attenuated GDC-0349-induced NSCLC cell death and apoptosis. Third, GDC-0349 was able to exert further cytotoxicity in Akt1-KO A549 cells, where Akt-mTOR activation was completely blocked. Indeed our results showed that GDC-0349 induced SphK1 inhibition, ceramide production, JNK activation and oxidative injury in NSCLC cells. SphK1 inhibition, JNK activation and oxidative stress were also detected in A549 xenograft tumors with GDC-0349 administration. These actions are clearly not the result of Akt-mTOR inhibition, as the mTOR inhibitor AZD-2014 or CRISPR/Cas9-induced Akt1 KO failed to affect SphK1 activity, JNK and ROS in NSCLC cells. These Akt-mTOR-independent mechanisms could explain the superior anti-NSCLC cell activity by this compound.

Sphingolipid metabolites, including S1P, ceramide, and sphingosine, are important players in the progression of human cancer^35^. S1P will promote cell survival and proliferation, while ceramide and sphingosine could induce cell growth arrest and apoptosis. SphK1, which catalyzes ceramide and sphingosine to S1P, is often overexpressed in NSCLC^33,57–59^ and other human cancers^35^, associated with tumorigenesis, cancer progression and resistance to therapies^33,35,57–59^. SphK1 inhibition will lead to ceramide (and sphingosine) accumulation, mediating cancer cell apoptosis^35^. In this study we show that GDC-0349 potently inhibited SphK1 activation, causing ceramide accumulation and JNK activation in NSCLC cells. Exogenously adding S1P alleviated GDC-0349-induced NSCLC cells death and apoptosis. These results indicating that SphK1 inhibition accounted, at least in part, for GDC-0349-induced anti-NSCLC activity. Although the underlying mechanisms may warrant further characterizations.

## Conclusion

Despite the latest developments in screening and early diagnosis of NSCLC, as well as the emerging of novel targeted therapies, the five-year overall survival for advanced NSCLC patients has only moderately improved over the past three decades^1,2^. Therefore, development of new therapeutic interventions for this devastating malignancy is urgently needed^3,60^. The results of this study demonstrated that GDC-0349 targeted multiple signaling cascades (Akt-mTOR and beyond) and potently inhibited NSCLC cell growth in vitro and in vitro. Therefore, it would be interesting to further testing this compound for NSCLC in future studies.
